# The Entangled Conductive Structure of CB/PA6/PP MFCs and Their Electromechanical Properties

**DOI:** 10.3390/polym13060961

**Published:** 2021-03-21

**Authors:** Yu Wang, Song Liu, Huihao Zhu, Huajian Ji, Guo Li, Zhou Wan, Yulu Ma, Linsheng Xie

**Affiliations:** School of Mechanical and Power Engineering, East China University of Science and Technology, Shanghai 200237, China; apirl.1992@outlook.com (Y.W.); lsezhou@163.com (S.L.); zhhxhhy1994@126.com (H.Z.); shxiaoji@163.com (H.J.); liguo119804@126.com (G.L.); wzhdlg@126.com (Z.W.); myl@ecust.edu.cn (Y.M.)

**Keywords:** microfibrillar composites (MFCs), conductive structure, electromechanical property, sensing

## Abstract

In this work, carbon black (CB)/polyamide 6 (PA6)/polypropylene (PP) microfibrillar composites (MFCs) were fabricated through an extrusion (hot stretching) heat treatment process. The CB-coated conductive PA6 microfibrils with high aspect ratio were in situ generated as a result of the selective accumulation of CB at the interface. At the proper temperature, a 3D entangled conductive structure was constructed in the PP matrix, due to topological entanglement between these conductive microfibrils. This unique conductive structure provided the PP composites with a low electrical conductivity percolation threshold. Moreover, the electromechanical properties of conductive MFCs were investigated for the first time. A great stability, a high sensitivity and a nice reproducibility were achieved simultaneously for CB/PA6/PP MFCs. This work provides a universal and low-cost method for the conductive polymer composites’ (CPCs) fabrication as sensing materials.

## 1. Introduction

Conductive polymer composites (CPCs) have already been applied extensively to many fields, such as antistatic materials [1], electromagnetic shield materials [2,3], and sensing materials [4,5,6] etc. Based on the resistance responses of CPCs to external stimuli, the sensing behavior of CPCs has received great attention, especially for their tensile-strain electrical-resistivity sensing behavior.

Conductive fillers are usually incorporated into the polymer matrix to fabricate CPCs with excellent strain sensing behavior [7,8,9]. Some studies found that the strain sensing behavior of CPCs depends strongly on the dimensionality of the conductive fillers [10]. For example, carbon black (CB) powders have the characteristics of low cost, high disorder and good conductivity, which could provide composites with excellent mechanical and electrical properties, and easy processability [11,12]. It was reported that the tensilestrain response behavior of CB/polydimethylsiloxane (PDMS) CPCs showed a higher sensitivity with a gauge factor (GF) of 15.75, compared with carbon nanotubes (CNTs)/PDMS with a GF of 4.36 [13]. However, during the tensile-retraction cyclic process, the resistance peak of CNT based CPCs usually remained unchanged or moved down gradually with an increase in the tensile cycle number, showing a higher stability when compared with a gradual increasing resistance peak of CB-based CPCs [13,14,15]. It is believed that this was due to the large aspect ratio and entanglement characteristics of CNTs [16,17].

However, high sensitivity, good stability and suitable electrical conductivity are required synchronously in CPCs as desirable sensing materials. In addition, it is difficult for CNTs to be used in large-scale applications, due to their high cost and complex preparation. Hence, some studies focused on regulating the conductive structure of CB/PP CPCs to satisfy the requirements of the sensing materials.

Fibrillar fillers with a large aspect ratio, such as glass fibers (GFs) [18], carbon fibers (CFs) [19], cellulose fibers [20], etc., have been added to CB/PP CPCs to construct a unique conductive structure. For example, Zhao et al. [21] incorporated CFs into CB/PP CPCs and prepared CFs/CB/PP CPCs. The CFs provided charge transport over long ranges, and bridged CB particles with long distances, which made CB transport electrons over short ranges. Hence, a bridged–segregated conductive structure was constructed in CFs/CB/PP CPCs, which endowed the composites with an improved sensitivity and a good stability during tensile–strain response. Wu et al. [22] prepared CB/natural rubber (NR) composites with a hierarchical conductive structure by the incorporation of cellulose nanowhiskers. The resultant CPCs showed a high liquid sensitivity, a good reproducibility and a low electrical conductivity percolation threshold. In addition, CNTs with large aspect ratios were also added to the CPCs to connect CB aggregates, and an excellent electromechanical performance was achieved in the composites [23,24]. However, as mentioned above, the cellulose nanowhiskers and CNTs, etc., nanofillers are difficult for large-scale industrial production, due to their poor dispersion and high cost. GFs and CFs, etc., traditional fibers have the characteristics of poor processability, and easy breakability during processing, which have a significantly negative influence on the comprehensive performance of CB-based CPCs.

In our previous study [25], the PA6 polymer fibrils were in situ generated in the PP matrix through an in situ fibrillation technique. These in situ generated fibrils had the characteristics of high aspect ratios and great dispersion [26,27]. Hence, it is obvious that they have more opportunities to balance the comprehensive performance of CPCs, compared with traditional fibers. Furthermore, although the temperature coefficient effect of conductive microfibrillar composites (MFCs) has been studied [28,29], to our knowledge, few studies focused on their electromechanical properties.

In this study, CB/PA6/PP MFCs were fabricated through a melt-extrusion hot-stretching heat-treatment process. At the proper temperature, an entangled conductive structure was constructed in the PP matrix. The CB/PA6/PP composites with different stretch ratios were also prepared for comparison. The morphology, electrical properties and electromechanical properties of these samples were investigated in detail.

## 2. Materials and Methods

### 2.1. Materials and Sample Preparation

The PP (tradename PF814) was purchased from Lyondellbasell Industries Corporation Ltd. (Hoofddorp, the Netherlands). Its melt flow rate is 2.5 g/10 min (230 °C, 2.16 kg, ASTM Standard D1238). The PA6 (tradename B27E) was purchased from BASF SE Corporation Ltd. (Shanghai, China). It has a density of 1.14 g/cm^3^ (ISO 1183), a melt flow rate of 130 cm^3^/10 min (275 °C, 5.0 kg, ISO 1133) and a melting temperature of 220 °C. CB (Printex XE2-B) was provided by Evonik Industries (Shanghai, China). It has a specific surface area of 1000 m^2^/g, a mean particle size of 35 nm, and a density of 2.13 g/cm^3^.

“Melt-extrusion”: CB and PP pellets were melt–blended by a two-rotor continuous extruder (*D* = 30 mm, *L/D* = 40, ECM30, homemade), and a CB/PP masterbatch was prepared. Then, CB/PA6/PP composites were prepared through mixing the CB/PP masterbatch and PA6 pellets. The screw speed was 500 rpm. The barrel temperature of the extruder was 50, 100, 150, 180, 200, 210, 220, 230, and 220 °C from the feed section to the die. The weight ratio of the CB/PP masterbatch and PA6 pellets is fixed at 85:15. The volume fraction of CB in the composites is 1, 3, 5, 7, 9 vol%.

“Hot-Stretching”: CB/PA6/PP blends were extruded by a single-screw extruder with a slit die (200 × 0.5 mm^2^). The screw speed was 40 rpm. The barrel temperature of the extruder was 180, 210, 230, 230, 230, 230, 230, 220 °C. The extrudates were stretched by a roller with a distance of 100 mm from the slit die exit. The roller speed was set as 0.3, 1.5, 3 and 4.5 m/min. According to the definition of stretch ratio (*λ*) [26], the corresponding stretch ratio was obtained as 1, 5, 10 and 15. After stretching, CB/PA6/PP film samples were shaped by a take-up roller.

“Hot-treatment”: By the chipping and compression molding process, the film samples were prepared into 130 × 130 × 0.5 mm^3^ samples at 200 °C for 10 min with a pressure of ca. 10 MPa.

In this paper, CB/PA6/PP composites with 3 vol% CB were chosen as the main research samples according to conductive percolation behavior.

### 2.2. Characterizations

Scanning electron microscopy (SEM): the phase morphology of the samples was observed by SEM. The samples were cryogenically fractured after placing in liquid nitrogen for 30 min. Some of the cryo-fractured samples were immersed in xylene for 5 h at 120 °C for dissolving away the PP. Others were used directly for the SEM observation. After covering with a thin layer of gold, these fractured surfaces were observed by the SEM (JSM-6360LV, Hitachi, Tokyo, Japan) device.

Transmission Electron Microscope (TEM): thin slices of the samples were observed by TEM (JEM-1400, UC7, Leica, Tokyo, Japan), and the elements of the polymer phase were analyzed by energy dispersive spectrometer (EDS, EDAX, Shanghai, China). At room temperature, the thin slices were cut by a diamond knife from the samples.

Electrical conductivity test: the electric conductivity of the samples was measured by a four probe digital meter (ST2253, Jingge Electronic Co., Ltd., Suzhou, China) for *R* < 10^5^ Ω or a high-resistance meter (ZC360, Shanghai, China) for *R* > 10^5^ Ω. The volume resistance *R_v_* (Ω) was transformed to volume resistivity *ρ_v_* and electrical conductivity *σ* using the following formula:

(1) $$\rho_{v}=R_{v}\frac{S}{d},\sigma =\frac{1}{\rho_{v}}$$

where *S* (m^2^) represents the effective area of the electrode and *d* (m) represents sample thickness. The electrically conductive percolation threshold could be predicted by an universal percolation law [30]:

(2) $$\sigma =\sigma_{0}{\left(\varphi -\varphi_{c}\right)}^{t}$$

where *σ* is the electrical conductivity of the CPCs, *σ*_0_ is the electrical conductivity of the conductive fillers, *φ* is the volume fraction of the conductive fillers, *φ_c_* is the electrically conductive percolation threshold, and *t* is a critical exponent.

Electro-mechanical property: a versatile testing machine (RGM-2020, Ruigeer Instrument Co., Ltd., Shenzhen, China) and an electrochemical workstation (CHI660E, Chenhua Instrument Co., Ltd., Shanghai, China) were used to test the electrical–mechanical response of the samples. The dimension of the samples was 100 × 10 × 0.5 mm^3^ and the gauge length was 40 mm. Silver paste was stuck onto the surface of the samples to guarantee fine electrical contact. Then these samples were subjected to monotonic tension-strain and ten cyclic tension-strain to 3% strain at a strain rate of 1 mm/min. The resistance was real-time recorded.

## 3. Results and Discussion

### 3.1. Morphology

**Figure 1:**
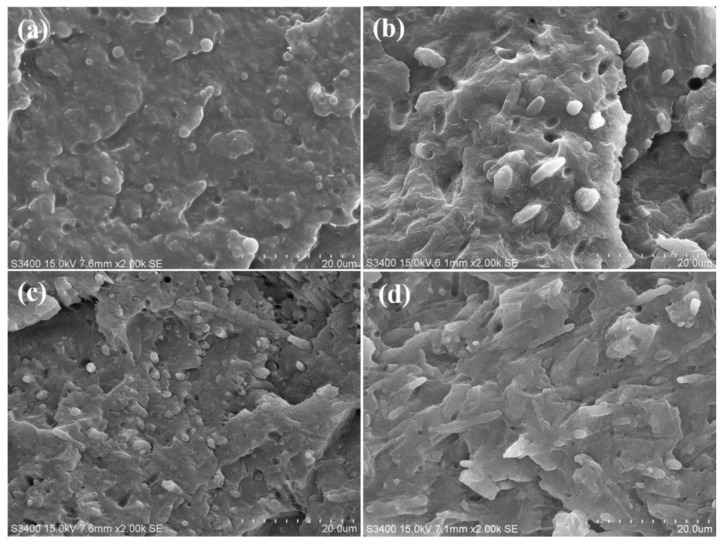
SEM micrographs of the carbon black (CB)/PA6/PP (3 vol% CB) composites with different stretch ratios: (**a**) *λ* = 1, (**b**) *λ* = 5, (**c**) *λ* = 10, (**d**) *λ* = 15.

The SEM micrographs of CB/PA6/PP (3 vol% CB) composites with different stretch ratios are shown in Figure 1. In terms of the stretch ratio of 1 (Figure 1a), the elongational flow is not considerable, and spherical PA6 phases are immersed in the PP matrix, indicating a typical two-phase incompatibility. At the stretch ratio of five (Figure 1b), a small amount of PA6 phases evolve into rod-like, and most remain spherical. When the stretch ratio is 10, the fibrillation of PA6 phases could be observed in Figure 1c, indicating that the dispersed phase morphology transferred from an isolated spherical domain to a completely fibrillated one. Especially for the stretch ratio of 15, the PA6 phases are fibrillated to a higher degree and form the microfibrils with a higher aspect ratio, as shown in Figure 1d. Hence, the samples with a high stretch ratio (10 and 15) are hereafter referred to as CB/PA6/PP MFCs.

**Figure 2:**
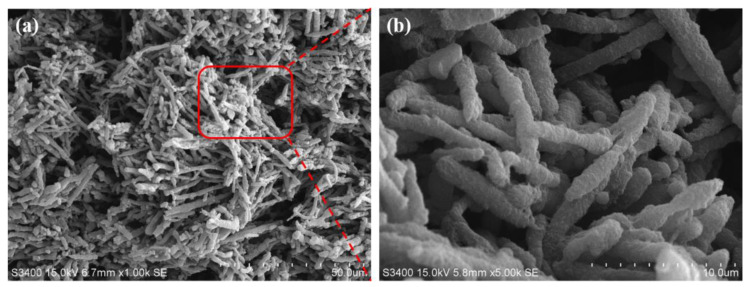
(**a**) SEM micrographs of the CB/PA6/PP MFCs after dissolving away PP, (**b**) SEM micrographs of red square region of Figure 2a at a higher magnification.

In order to observe the phase morphology more clearly, Figure 2 shows SEM micrographs of CB/PA6/PP MFCs after dissolving away the PP matrix. An entangled structure is observed, consisting of the topological interaction between PA6 microfibrils. This is because, at a proper temperature (between the melting temperature of PP and PA6), phase morphology of PA6 microfibrils could be retained, and these microfibrils connected with each other, caused by local flow and diffusion of the melted PP. At a higher magnification (Figure 2b), the rough surfaces of the PA6 microfibrils are observed, which is different from the smooth surfaces of the dispersed phase in PP/PA6 MFCs, as shown in Figure S1. This might be due to the accumulation of CB at the interface, and more qualitative analysis is needed.

**Figure 3:**
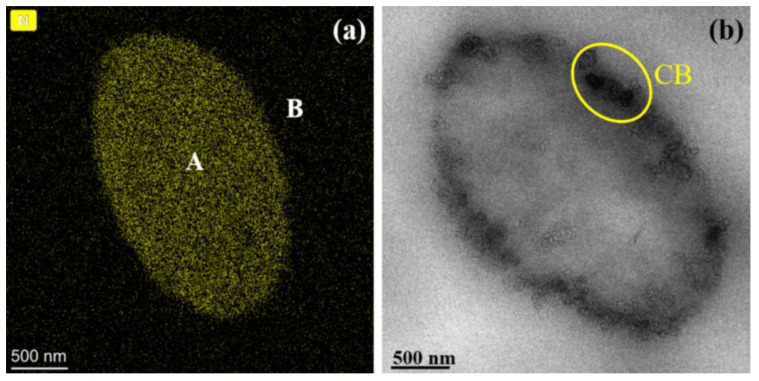
(**a**) EDS mapping image of the elements N (yellow) and (**b**) TEM micrograph of the cross-section of individual PA6 microfibrils surrounded with the PP matrix.

Figure 3 shows the EDS mapping image and TEM micrograph of the cross-section of individual PA6 microfibrils surrounded with the PP matrix. The yellow dots represent the elements N in the EDS mapping image. These yellow dots coalesce in the A region, and few are in the B region, indicating that the A region is the PA6 phase, and an obvious phase separation is observed. Moreover, a large amount of CB aggregates accumulates on the PA6 phase side of the interface, and a small amount of CB is located inside the PA6 phase, as show in Figure 3b. These results demonstrate that CB migrates from the PP to PA6 phase, and is distributed at the phase interface.

The migration of CB in immiscible polymer blends could be estimated by the wetting coefficient *ɷ_a_* [31]:

(3) $$\omega_{a}=\frac{\gamma_{CB-PP}-\gamma_{CB-PA6}}{\gamma_{PP-PA6}}$$

where *ɷ_a_* represents the wetting coefficient and *γ* represents the interfacial tension. When *ɷ_a_* > 1, the CB particles prefer to accumulate in the PA6 phase; when −1 < *ɷ_a_* < 1, the CB particles prefer to accumulate at the interface, and when *ɷ_a_* < −1, the CB particles prefer to accumulate in the PP phase. The interfacial tension between the two phases could be estimated using the geometric–mean equation and the harmonic–mean equation [32]:

(4) $$\gamma_{AB}=\gamma_{A}+\gamma_{B}-2{\left(\gamma_{A}^{d}\gamma_{B}^{d}\right)}^{1/2}-2{\left(\gamma_{A}^{p}\gamma_{B}^{p}\right)}^{1/2}$$

(5) $$\gamma_{AB}=\gamma_{A}+\gamma_{B}-4(\frac{\gamma_{A}^{d}\gamma_{B}^{d}}{\gamma_{A}^{d}+\gamma_{B}^{d}}+\frac{\gamma_{A}^{p}\gamma_{B}^{p}}{\gamma_{A}^{p}+\gamma_{B}^{p}})$$

where *γ_i_* represents the surface tension of component *i*, and *γ^d^* and *γ^p^* represent the dispersive and polar contributions, respectively. The surface tensions (230 °C) [33] of PP, PA6 and CB are shown in Table 1. The calculated interfacial tension and the wetting coefficient are shown in Table 2. According to Equations (4) and (5), *ɷ_a_* equals to 3.48 and 4.01. This means that the CB has a higher affinity towards PA6 than PP phases. The CB/PP masterbatch method made CB aggregates migrate from PP to PA6. Smaller CB aggregates preferred to migrate into the inside of the PA6 phase, while larger CB aggregates were trapped at the interface region, and formed continuous conductive pathways [34]. Hence, the conductive PA6 microfibrils were in situ generated in the PP matrix.

**Figure 4:**
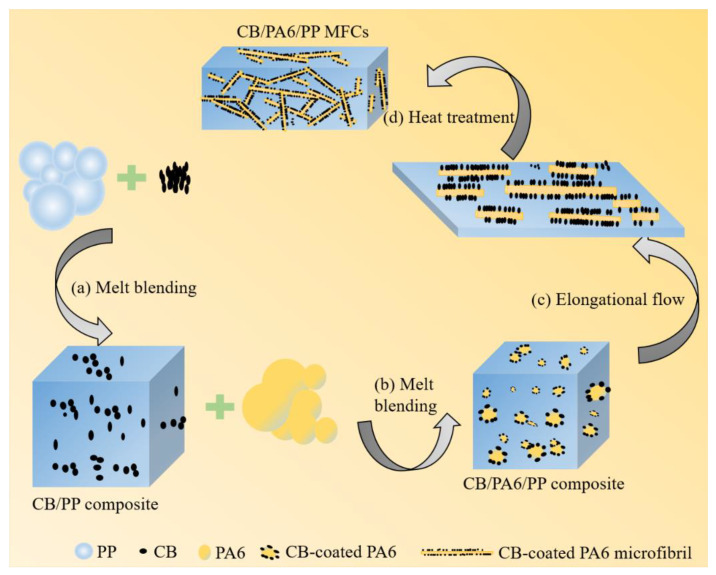
The formation diagram of the conductive network of the CB/PA6/PP microfibrillar composites (MFCs), (**a**) Melt bending; (**b**) Melt bending; (**c**) Elongational flow; (**d**) Heat treatment.

Due to the synergistic effect of the fine connection between PA6 microfibrils and the selective accumulation of CB at the interface, a 3D entangled conductive structure consisting of CB-coated PA6 microfibrils is formed in the CB/PA6/PP MFCs. The formation diagram is shown in Figure 4. CB and PP are firstly mixed, and the CB/PP masterbatch is prepared (Figure 4a). Then, PA6 is added to the CB/PP masterbatch, and CB/PA6/PP blends are prepared. During this process, CB particles migrate from PP to PA6, and accumulate at the interface region (Figure 4b). The CB/PA6/PP blends are extruded through a slit die. Under the combined effect of the elongational and shear flow, CB-coated PA6 microfibrils are in situ generated in the PP matrix and aligned with the stretch direction (Figure 4c). During the following compression mold, local flow and diffusion of the melted PP make CB-coated PA6 microfibrils interconnect with each other and form a 3D entangled conductive structure (Figure 4d).

### 3.2. Electrically Conductive Properties

**Figure 5:**
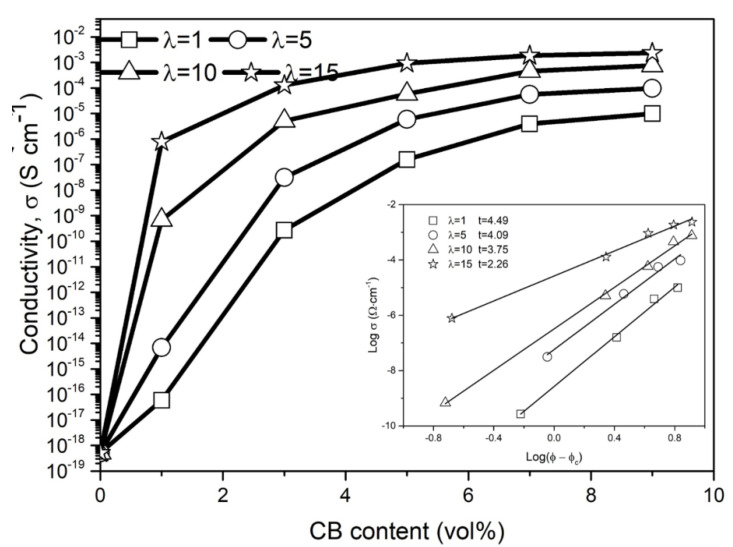
Electrical conductivity of the CB/PA6/PP composites with CB content; the inset is the log(*φ − φ_c_*) vs. log *σ* plot.

The variations of the electrical conductivity of CB/PA6/PP composites with CB content are shown in Figure 5. A fit of log *σ* vs. log *(φ − φ_c_*) is shown in the inset of Figure 5. There is a significant jump in the electrical conductivity, when the CB content is higher than a critical value, indicating percolation behavior of the CPCs [35]. The critical value is generally called the electrically conductive percolation threshold. The electrically conductive percolation threshold *φ_c_* of CB/PA6/PP composites could be estimated as 2.43, 2.14, 0.83 and 0.79 vol% according to Equation (2), respectively, corresponding to the stretch ratio of 1, 5, 10 and 15. Obviously, as the stretch ratio increases, the percolation threshold of the CB/PA6/PP composites decreases gradually. This indicates that it was more perfect for the conductive structure of CB/PA6/PP MFCs. The decrease of the percolation threshold is determined largely by different microstructures, as shown in Section 3.1. For the composites with a low stretch ratio (*λ* = 1 and 5), CB-coated PA6 phases were spherical and dispersed as many separate islands in the PP matrix. The electrons could only be transferred through building tunneling pathways [36]. It was difficult to form the conductive channels. Hence, more CB is required to achieve a conductive percolation behavior. However, CB-coated PA6 microfibrils entangled topologically in the CB/PA6/PP MFCs. The construction of the conductive channels was mainly through the interconnection between these microfibrils. Therefore, it was easier to build more conductive channels and construct a more perfect conductive structure, which led to a lower electrically conductive percolation threshold.

The t of Equation (2) could be used to further describe the formation mechanism of a different conductive structure. When the *t* value is between 1.1 and 1.3, the conductive structure is a two-dimensional (2D) system; when the t value is between 1.6 and 2.0, it is a three-dimensional (3D) system [36,37]. As shown in the inset of Figure 5, the *t* value of CB/PA6/PP composites decreases from 4.49 to 2.26 with the stretch ratio increases. Although all *t* values are out of the range, some reports have proved that the practical conductive systems always deviate from the universal percolation law [38]. But it could be confirmed that more contact conduction led the *t* value to be smaller, and more tunneling conduction led the *t* value to be larger [28]. This result is in accordance with the above analysis.

### 3.3. Electromechanical Properties

#### 3.3.1. Electromechanical Properties under Uniaxial Tension-Strain

**Figure 6:**
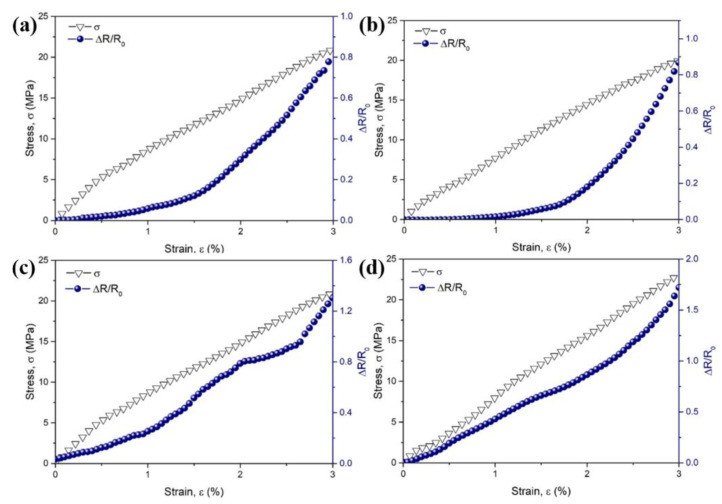
Stress and Δ*R/R_0_* as a function of tensile strain for CB/PA6/PP composites: (**a**) *λ* = 1, (**b**) *λ* = 5, (**c**) *λ* = 10, (**d**) *λ* = 15 under the uniaxial tension-strain.

The stress–strain and Δ*R/R_0_*–strain curves for the CB/PA6/PP composites with different stretch ratios under uniaxial tension-strain are shown in Figure 6. Here, Δ*R* and *R_0_* represent the instantaneous resistance change and the initial resistance of the samples, respectively. The stress increases gradually with the increasing strain, and all samples show similar shapes of the stress–strain curves, indicating a typical brittle fracture. However, the Δ*R/R_0_*–strain curves of the composites have different trends. For the composites with a low stretch ratio (*λ* = 1 and 5), the Δ*R/R_0_* increases slightly at a small strain below 1%. With the further increasing strain, the Δ*R/R_0_* increases exponentially, as shown in Figure 6a,b. At this time, the breakdown of the conductive pathway is more dominant than the formation. This could be described by the Balberg Equation [37]:

(6) $$\sigma \propto \exp[-\frac{r-2b}{d}]$$

where *σ* represents the tunneling conductivity, *r* represents the center distance between the conductive particles, *b* represents the radius of the conductive particles, and *d* represents the typical tunneling range parameter. When tension-strain was applied, the position of the CB particles was changed gradually. A small strain made the center distance *r* between the CB displace in a small range, leading to a slight decrease in the tunneling conductivity *σ*, i.e., a little increase in the Δ*R/R_0_*. The further increase of the strain made the center distance *r* grow significantly, and the tunneling conductivity *σ* decrease exponentially, i.e., the Δ*R/R_0_* increased rapidly in an exponential trend.

In terms of the CB/PA6/PP MFCs, the Δ*R/R_0_* increases almost linearly with the increasing strain, as shown in Figure 6c,d. This indicates that the entangled conductive structure is extremely stable [10,14]; it is due to this that, when external force is applied, the entangled conductive structure consisting of CB-coated PA6 microfibrils had the features of storing deformation energy, which could balance the breakdown and formation of the conductive pathways more effectively. Hence, CB/PA6/PP MFCs showed a great stability under uniaxial tension-strain.

In addition, it could be seen that the Δ*R/R_0_* of CB/PA6/PP MFCs increases much faster, indicating a higher sensitivity. The gauge factor (GF) [39] could be used to evaluate the sensitivity of the CPCs under tension-strain.

(7) $$GF=\frac{\Delta R/R_{0}}{\varepsilon }$$

where Δ*R* represents the instantaneous resistance change, *R_0_* represents the initial resistance, and *ε* represents the tensile strain. The variations of gauge factors with tension-strain for the composites with different stretch ratios are shown in Figure S2. At the strain of 3%, GF could be evaluated as 69.2, 99.3, 101.0 and 201.6 for the composites with stretch ratios of 1, 5, 10 and 15. This is because the accumulation of CB at the interface raised the sensitivity to tensile stimuli. Moreover, the contact junctions between CB-coated PA6 microfibrils were easier to be disrupted, leading to higher sensitivity of the CB/PA6/PP MFCs under uniaxial tension-strain.

#### 3.3.2. Electromechanical Properties under Cyclic Tension-Strain

**Figure 7:**
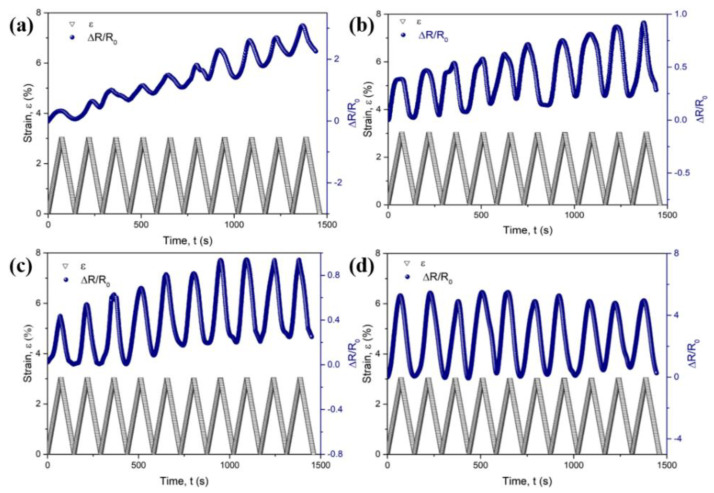
The variations of strain and Δ*R/R_0_* with time for CB/PA6/PP composites: (**a**) *λ* = 1, (**b**) *λ* = 5, (**c**) *λ* = 10, (**d**) *λ* = 15 in ten times extension–retraction cycles.

The ten times cyclic curves of strain–time and Δ*R/R_0_*–time are shown in Figure 7. The Δ*R/R_0_* of all samples increases with the loading strain and decreases with the unloading strain in each cycle. This is because in the tension-strain process, CB-coated PA6 phases deformed with the PP matrix due to the viscoelastic characteristics, leading to the change in the CB position and the increase in the center distance *r*. The previously existing conductive pathways were deteriorated, causing Δ*R/R_0_* to gradually increase. While in the retraction process, the deformation of the PP matrix recovered, causing the decrease in the center distance *r* of CB, and the reconstruction of the new conductive pathways. Hence, the Δ*R/R_0_* had a gradual decrease.

**Figure 8:**
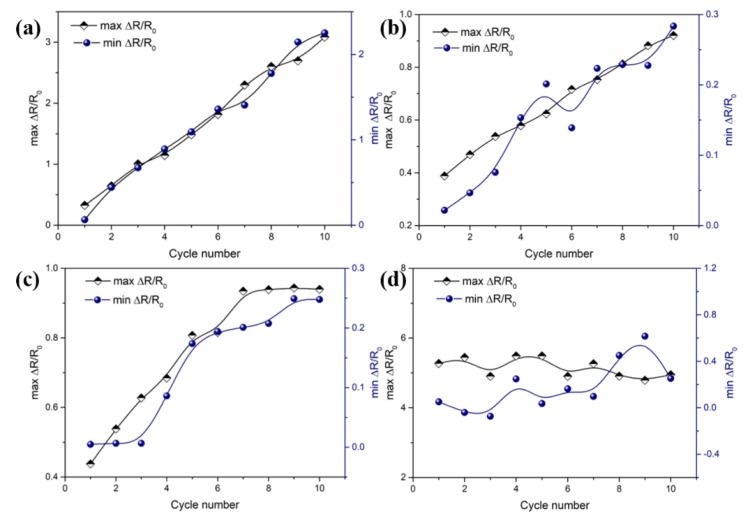
The variations of max Δ*R/R_0_* and min Δ*R/R_0_* with cycle number for CB/PA6/PP composites: (**a**) *λ* = 1, (**b**) *λ* = 5, (**c**) *λ* = 10, (**d**) *λ* = 15 in ten times extension–retraction cycles.

It is noteworthy that the variations of max and min Δ*R/R_0_* in the ten times cyclic tension show a remarkable difference for the composites with different stretch ratios, as shown in Figure 7. The relationship of max and min Δ*R/R_0_* with the cycle number is shown in Figure 8. For the CB/PA6/PP composites with a low stretch ratio (*λ* = 1 and 5), the max and min Δ*R/R_0_* move up gradually with the increasing cycle number, as shown in Figure 8a,b. A gradual increase in min Δ*R/R_0_* is due to the viscoelastic effects of the PP matrix which led to the irreversible changes of the CB position at the end of every cycle. The conductive paths have been deteriorated in the tension process and could not be fully recovered in the retraction process. Hence, the min Δ*R/R_0_* of the composites increases gradually with the increasing cycle number. In the following tension process, the destroyed conductive paths were further deteriorated, causing a gradual increase in the max Δ*R/R_0_*.

The difference is that, after the six times extension–retraction cycles, max and min Δ*R/R_0_* reach a stable value for the composites with a stretch ratio of 10, as shown in Figure 8c. Especially for the composites with the stretch ratio of 15, the max and min Δ*R/R_0_* keep at around 5 and 0.2 throughout the cycles, as shown in Figure 8d. These results indicate that the CB/PA6/PP MFCs had a nice reproducibility under cyclic tension. During the ten times extension–retraction cycles, although the CB position was rearranged all the time, CB-coated PA6 microfibrils always interconnected with each other, leading to the conductive pathways being rebuilt constantly. Moreover, the damage degree of the conductive pathways was reduced greatly due to the entanglement of the microfibrils. Hence, a stable max and min Δ*R/R_0_* was achieved for CB/PA6/PP MFCs.

#### 3.3.3. Mechanism Analysis

**Figure 9:**
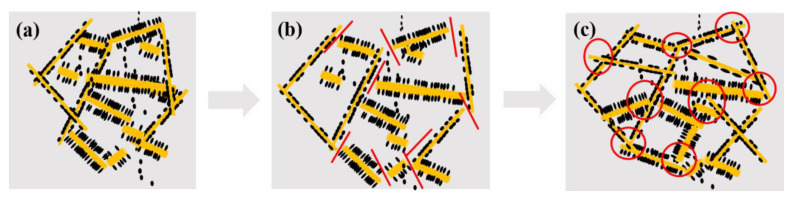
Schematic illustration of the conductive network evolution of CB/PA6/PP MFCs: (**a**) initial conductive network; (**b**) the conductive network under tension-strain; and (**c**) final conductive network. The lines and circles demonstrate the destruction and rebuilding of the conductive paths, respectively.

A schematic illustration of the conductive structure evolution for CB/PA6/PP MFCs in the extension–retraction process is shown in Figure 9. An initial conductive network before tension is shown in Figure 9a. It can be seen that there is the formation of a 3D entangled conductive structure in the PP matrix, which consists of the CB-coated PA6 microfibrils. When the tension strain is applied, the PP matrix deforms and the interfacial stress is transferred to the CB-coated PA6 microfibrils, then the initial conductive structure is destroyed, as shown in Figure 9b. At this time, it is more significant for the breakdown of the conductive pathways, as indicated by the red lines. In the following retraction process, CB-coated PA6 microfibrils could interconnect with each other again due to the recovery of the PP matrix, leading to the rebuilding of new conductive pathways (as indicated by the red circles) and the formation of a new conductive structure, as shown in Figure 9c. The SEM micrograph of the samples after electromechanical testing is shown in Figure S3. It can be seen that a new entangled conductive structure consisting of CB-coated PA6 microfibrils was rebuilt in CB/PA6/PP MFCs, which supports the above analysis well.

## 4. Conclusions

In this work, the CB/PA6/PP MFCs were fabricated through the extrusion hot-stretching heat-treatment process. A 3D entangled conductive structure was constructed in the CB/PA6/PP MFCs, due to selective accumulation of CB at the interface and the high aspect ratio of PA6 microfibrils. This unique conductive structure provided a low threshold percolation for PP composites due to topological interaction between CB-coated conductive PA6 microfibrils. Great stability, high sensitivity and nice reproducibility were achieved simultaneously for the electromechanical properties of the MFCs. This work offers a large-scale preparation method of CPCs as an application for sensing materials.

## Figures and Tables

**Table 1:** Surface tension of PP, PA6 and carbon black (CB) at 230 °C [33].

Component	Surface Tension *γ* (mN/m)	Dispersive Surface Energy *γ^d^* (mN/m)	Polar Surface Energy *γ^p^* (mN/m)
CB	85.50	82.36	3.14
PA6	37.19	35.00	2.19
PP	17.62	17.25	0.37

**Table 2:** The calculated interfacial tension and wetting coefficient at 230 °C.

Equation	*γ*_CB-PP_ (mN/m)	*γ*_CB-PA6_ (mN/m)	*γ*_PP-PA*6*_ (mN/m)	*ɷ_a_*
Geometric–mean equation	19.28	44.75	7.32	3.48
Harmonic–mean equation	10.07	25.58	3.87	4.01

## Supplementary Materials

The following are available online at https://www.mdpi.com/2073-4360/13/6/961/s1. Figure S1: SEM micrographs of PP/PA6 MFCs. Figure S2: the variations of gauge factors with strain for the CB/PA6/PP composites with different stretch ratios. Figure S3: SEM micrograph of CB/PA6/PP MFCs after electromechanical testing.
