# Supplementary material for: The Entangled Conductive Structure of CB/PA6/PP MFCs and Their Electromechanical Properties
Source: Polymers (Basel). 2021 Mar 21;13(6):961. doi: 10.3390/polym13060961 (PMC8003847; doi:10.3390/polym13060961)
Supplement: Supplementary file 1 [file polymers-13-00961-s001.pdf]

# Supplementary Materials: The entangled conductive structure of CB/PA6/PP MFCs and their electromechanical properties

**Yu Wang, Song Liu, Huihao Zhu, Huajian Ji, Guo Li, Zhou Wan, Yulu Ma, and Linsheng Xie***

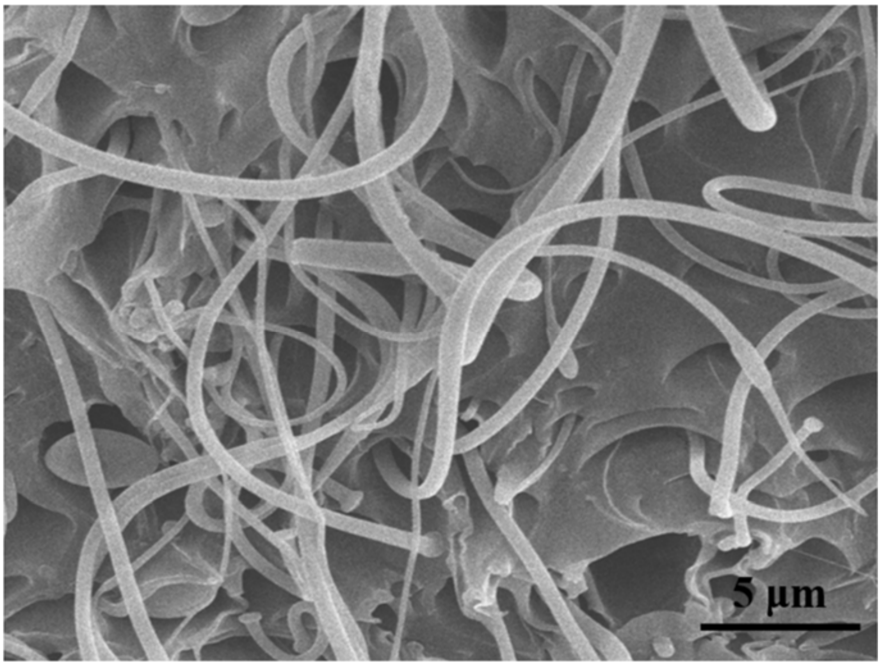


**Figure S1.** SEM micrographs of PP/PA6 MFCs.

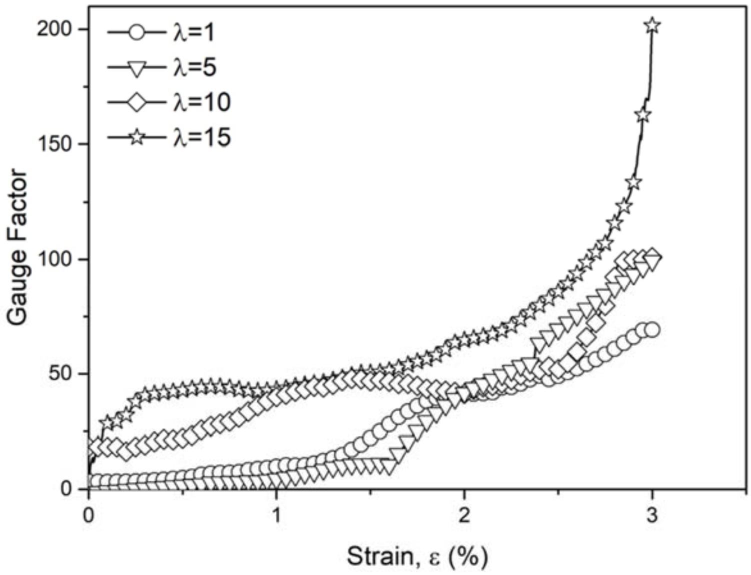


**Figure S2.** The variations of gauge factor with strain for the CB/PA6/PP composites with different stretch ratios.

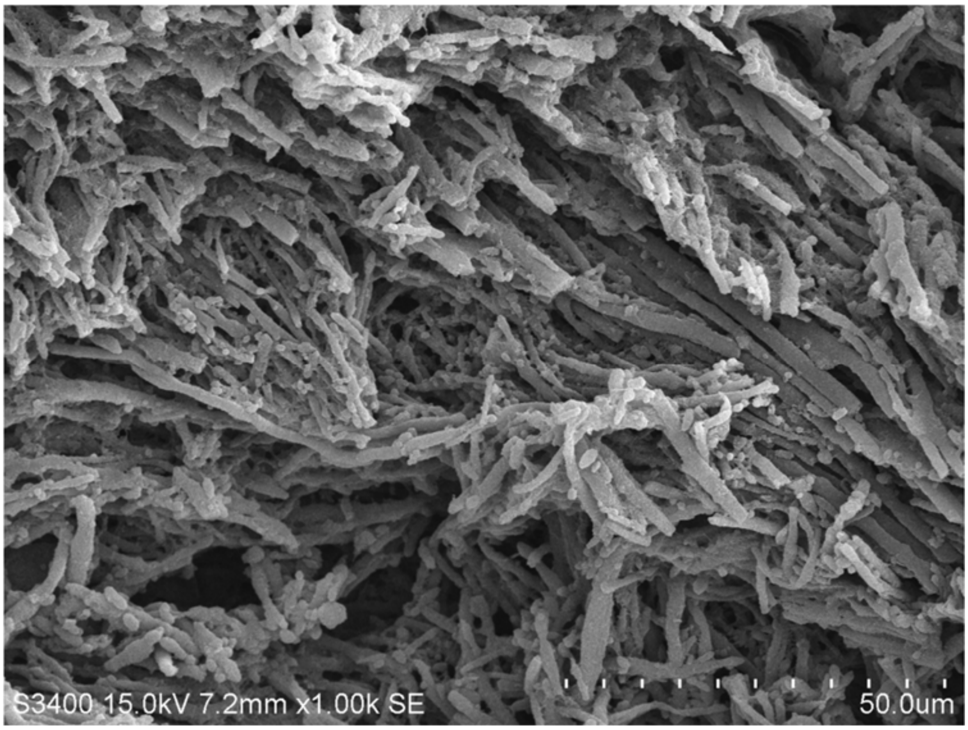


**Figure S3.** SEM micrograph of CB/PA6/PP MFCs after electromechanical testing.
